# Hand posture, but not vision of the hand, affects tactile spatial resolution in the grating orientation discrimination task

**DOI:** 10.1007/s00221-022-06450-3

**Published:** 2022-09-08

**Authors:** B. French, N. V. Di Chiaro, N. P. Holmes

**Affiliations:** School of Psychology, University of Nottingham, Nottingham, UK

**Keywords:** Tactile spatial perception, Grating orientation task, Psychophysics

## Abstract

The grating orientation discrimination task (GOT) is a sensitive and reliable measure of tactile spatial resolution, or acuity. We used the GOT in three experiments to investigate the effects of hand posture and hand visibility on spatial acuity. Participant sex and experimental design were also manipulated. Healthy adult participants received brief touches to their index fingertips of grated, domed objects. Their task was to decide whether the gratings ran 'along' or 'across' their finger. Measures of the smallest grating width for which participants could reliably discriminate between orientations were recorded as threshold. Experiment 1 evaluated the effect of two- versus one-interval discrimination, hand used and participant sex. Experiments 2 and 3 evaluated the effects of hand visibility (visible or covered) and hand posture (in front or to the side). Females were better than males; the two-interval task resulted in lower thresholds than the one-interval task; and left and right hand thresholds were not significantly different. Most importantly, while hand visibility did not have a significant effect on the task, thresholds were affected by hand posture—worse when the hand was oriented to the side of the body than in front. These results replicate previously reported effects of sex (or finger size), but failed to replicate the so-called ‘visual enhancement of touch’ (VET) effect. We also report a meta-analysis of 27 VET studies, finding a significant effect of ‘non-informative’ vision on tactile perception. Our novel finding is that hand posture affects tactile acuity.

**Supplementary Information:**

The online version contains supplementary material available at 10.1007/s00221-022-06450-3.

## Introduction

Our ability to discriminate the fine spatial details of objects touching our body passively—tactile ‘spatial resolution’ or tactile acuity—can be estimated using the grating orientation discrimination task (GOT; Craig 1999; Johnson and Phillips 1981; see Ryan et al. 2021 for a review of tactile texture perception). The grating orientation task involves presenting curved surfaces with varying widths of square-wave gratings onto the skin, for example across or along a fingertip. The ability to discriminate the orientation of the gratings provides a reliable measure of tactile spatial resolution. On the human fingertip, the orientation of gratings as narrow as 1 mm can be discriminated. Tactile spatial resolution on the fingertip worsens with age (Manning and Tremblay 2006) and is better in females, but this effect of sex is entirely explained by the size of participants’ fingers (Peters et al. 2009). Women tend to have smaller fingers as well as a higher density of receptors in the ridges of the fingertip (Dillon et al. 2001; Jarocka et al. 2021).

Tactile spatial resolution may be affected by manipulating non-tactile sources of information. For example, a 'visual enhancement of touch' (VET) effect has been reported as an improvement in tactile perception while participants are able to view the body part being touched, compared to when viewing a neutral object at the same location (Taylor-Clarke et al. 2004). In the experimental condition of the VET, participants view their hand or other body part, but this vision of the body provides no information about the orientation of the grating or the time of tactile stimulation—so-called ‘non-informative’ vision. In the control condition, participants view a non-body object such as a tube or wooden block appearing at the same location. Several studies have reported that tactile performance is significantly better in the body condition than the object condition (Kennett et al. 2001; Taylor-Clarke et al. 2004). It has been argued that this is due to visual inputs modulating activity in the primary somatosensory cortex (Cardini et al. 2011). After a systematic review and meta-analysis of ten VET studies, Eads and colleagues (2015) indeed found better tactile performance when vision of the stimulated body part, particularly the hand, was available than when it was not. This suggests that non-informative vision of the body has a positive effect on tactile spatial perception across studies (although we provide a critique of this previous meta-analysis here).

The present study addressed three aims across three experiments and a meta-analysis. First, we aimed to validate our custom experimental stimuli and robotic manipulator by repeatedly measuring participants’ thresholds across multiple conditions, and to replicate well-known effects of participant sex and experimental design. We predicted that women should perform better than men, and that performance in a two-interval forced-choice design should be better than in a one-interval design. Second, we reasoned that manipulating both the visibility and the posture of the arm were likely to have effects on GOT performance. Following previous reports of the VET, we expected thresholds to be lower (better) when the hand was visible than when it was not. Following previous work on postural effects on bodily perception (see reviews in Holmes 2006, 2013), and our intuition that the GOT is performed partly using visuospatial imagery, we expected that thresholds would be lower when the hand was aligned with the head, body, and the direction of the stimuli than when it was misaligned. Third, we repeated and expanded upon a previous meta-analysis (Eads et al. 2015) to assess the evidence for the VET more broadly.

## Methods

### Participants

The experimental procedures were approved by the University of Reading Ethics Committee (UREC 14/09). Participants (Table 1) gave written, informed consent. One participant’s data were removed from Experiment 2 because they performed at chance level (threshold > 3 mm) on six of the eight experimental blocks. One participant’s data was removed from Experiment 3 because they performed almost perfectly, resulting in (impossible!) grating thresholds below 0.35 mm. We assume that this participant removed their finger from the stimulus aperture and performed the discrimination task visually.

**Table 1:** Experimental design and participants

E	*N*	*n*	F	M	R	Age in years range, mean (SD)	Presentation method	Design	Repeats
1	20	20	10	10	18	19–59, 31.2 (9.97)	Manual	Intervals (1, 2) x Hand (L, R) x Sex (F, M)	1
2	14	13	10	3	10	19–48, 25.5 (7.28)	Robot	Visibility (V, H) x Posture (A, S)	2
3	18	17	12	5	16	18–61, 31.9 (12.20)	Robot	Posture (A, S)	3

*E* experiment, *N* participants tested, *n* participants’ data included, *F* number of females, *M* number of males, *R* number of right-handed (by self-report), *L* left, *R* right, *F* Female, *M* male, *V* visible, *H* hidden, *A* ahead, *S* side

### Apparatus and materials

**Fig. 1:**
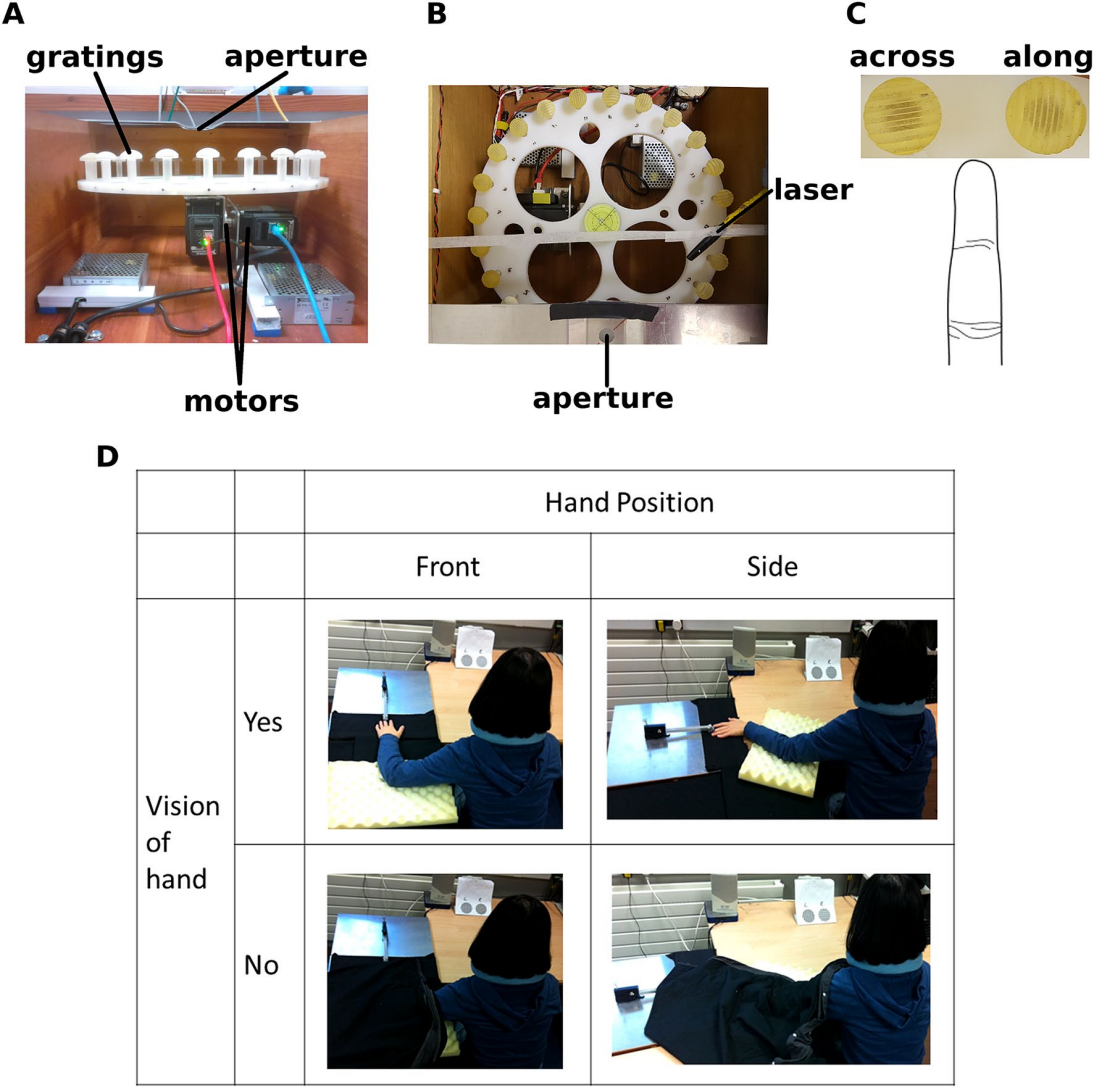
Grating orientation discrimination task (GOT) and apparatus. **A** Wooden box (45 cm wide, 50 cm deep, 25 cm high) housing two ethernet-controlled stepper motors, an acrylic disc (38 cm diameter) holding 12 × 2 3D-printed plastic domed gratings with ridge widths of 0.35, 0.5, 0.75, 1.0, 1.25, 1.5, 1.75, 2.0, 2.25, 2.5, 2.75, and 3.0 mm and diameter 2.5 cm at their widest point. **B** Top view of the grating wheel. Gratings were arranged in ascending order of grating widths, alternating across and along (clockwise from ~ 7 o’clock in Fig. 1B). A laser pointer was used as a light gate timing signal. **C** Two gratings photographed in position on the wheel next to a schematic finger at approximately the same scale. When rotated into position, the gratings run across and along the long axis of the finger. **D** In Experiments 2 and 3, hand position and hand visibility were manipulated

*Gratings:* 24 plastic domes, 2 each of 12 different grating widths (Fig. 1A; 0.35, 0.50, 0.75, 1.00, 1.25, 1.50, 1.75, 2.00, 2.25, 2.50, 2.75, 3.00 mm) were 3D printed at ShapeWays.com. The highest-spatial frequency gratings (0.35 mm) were printed using a different, finer, material from the others, but these gratings were very rarely used, as most participants’ thresholds were well above 0.5 mm.

*Grating presentation*: In Experiment 1, gratings were presented by hand. The experimenter pressed the grating into the participant’s finger pad through a 16 mm aperture in an aluminium plate to shield view of the grating from the participant. In Experiments 2, and 3, the gratings were presented automatically using a custom-built robot with two single-axis motors, one that rotated and one that tilted an acrylic wheel containing the 24 gratings: all 12 widths and each at two orientations, horizontal or vertical (Fig. 1A–C). Participants responded using two pedals, one under each foot.

### Design

This study aimed to test some basic manipulations of the GOT, and to replicate the VET effect. The design for each experiment is in Table 1. Experiment 1 explored the effect of experimental design, specifically one or two-interval forced choice (1-IFC vs. 2-IFC), and participant sex, on GOT performance. Experiments 2 and 3 tested the effects of hand visibility and hand posture. Conditions were created by combining the experimental variables factorially. Each condition was repeated one, two or three times, and run in fully counterbalanced order across participants.

Experiment 1 included four conditions, comprising the left or right hand with a single or two-interval design. Ten (self-identifying) female and ten male participants were recruited. We hypothesised that performance would be better for 2-IFC than 1-IFC, and that females would perform better on the GOT than males, because of their smaller hand size. Experiment 2 explored the effect of hand visibility (hand visible or not) and hand position (in front or to the side) on the GOT. A one-interval (horizontal vs. vertical) design was used. Following reports of the VET, it was expected that covering the hand of the participant would worsen performance on the GOT. Experiment 3 attempted to replicate the effect of hand position (ahead or to the side). Following Experiment 2, we expected to find higher thresholds when the hand was positioned to the side than in front. We did not repeat the visibility manipulation, and instead increased the number of repetitions.

### Procedure

In Experiment 1, the experimenter manually applied grated domes to the index fingertip of the participant (either vertically or horizontally), through a circular aperture in an aluminium plate, on which participants rested their hand. The participants decided whether the gratings ran 'along' (vertical, parallel with the long axis of the finger) or 'across' (horizontal, perpendicular to the long axis of the finger) their finger (1-IFC), or identified the interval in which the horizontal stimulus was presented (first or second). In 1-IFC trials, participants were given a single choice of whether the presented grating was vertical or horizontal. For 2-IFC blocks, each trial contained two temporal intervals of 1 s, and within each interval either a horizontal or vertical grating was presented to the participant. The two intervals were separated by a 2.5 s pause. The participants responded by releasing the left pedal for ‘across’ or ‘first interval’ and the right pedal for ‘along’ or ‘second interval’.

The grating width presented on each trial was determined by the QUEST staircase procedure in PsychToolbox 3 running in MATLAB (Watson & Pelli 1983, http://psychtoolbox.org/docs/Quest). QUEST was initialised with a mean ± SD threshold of 2 ± 2 mm, 76% correct at threshold, and the Weibull distribution parameters of beta = 3.5 (slope), delta = 0.05 (proportion ‘blind’ responses), and gamma = 0.5 (chance level); grain was set to 0.005, and range 4 in pilot testing. At the start of each trial, the next grating was chosen using QuestQuantile, rounded to the nearest-available grating width. The orientation was chosen pseudorandomly, with half of the trials requiring a left pedal response and half a right. In Experiment 1, this information was presented discretely to the experimenter on screen. In Experiments 2 and 3, it was used by the MATLAB script to control the robot. At the end of each trial, QUEST was updated using the presented grating width and a 0 for incorrect and 1 for correct, based on the participant’s response. Some participants performed a version of the experiment with 50 trials per staircase, but all thresholds reported here were based on the first 45 trials.

In Experiment 2, participants sat with their left hand in front of their body, (the forward or ‘ahead’ posture), or the robot was rotated and participants held their hand in a lateral posture (Fig. 1D). In each posture, the hand was either covered with a cloth, or kept visible. Grating presentation was automated by the robot. Each grating stimulus was presented to the finger for around 350 ms, based on recordings made later, in 2021, using a force sensor and an electronic light gate. Gratings were presented through an 18 mm aperture in an aluminium plate on top of the robot apparatus. Experiment 3 followed a very similar process to Experiment 2, but without the hand being covered. There were three trials for both hand levels (forward and lateral).

### Analysis

All experimental blocks resulted in a grating width discrimination threshold estimated by QUEST. This corresponded approximately to 76% correct performance. These thresholds were averaged across repetitions and analysed using within-participants and between-participants *t* tests in MATLAB to determine the effects of sex, design, hand position, and visibility on GOT performance. All data are reported as mean (M), standard deviation (SD), and standard error (SE), to allow easier meta-analyses in future.

### Meta-analysis

Prompted by a reviewer, we repeated and expanded upon the meta-analysis reported by Eads et al. (2015). We started with the ten papers included in that study and checked all the references to and from each of those ten papers, as well as to studies that cited the previous meta-analysis. We did not perform a systematic review, but found a total of 27 relevant studies including 50 independent effect sizes. Inclusion criteria were studies of healthy adult humans that included at least two conditions of a tactile task—one with vision of the body, and one without. We computed, and estimated where necessary, the standardised mean differences (Cohen’s d) from the reported statistics and graphical results in the relevant papers. Seven sets of paired, dependent effect sizes from the same studies were averaged to give a single Cohen’s d per independent group. Three of the 50 independent effects did not come with sufficient information to calculate Cohen’s d. A random-effects meta-analysis in JASP 0.9.2 was used to compute meta-analytic effect sizes on Cohen’s d. The full analysis and dataset are included as a supplementary spreadsheet and on our Open Science Framework project page (https://osf.io/da893/).

## Results

### Experiment 1: better performance for 2-IFC and by females, but no effect of hand

**Fig. 2:**
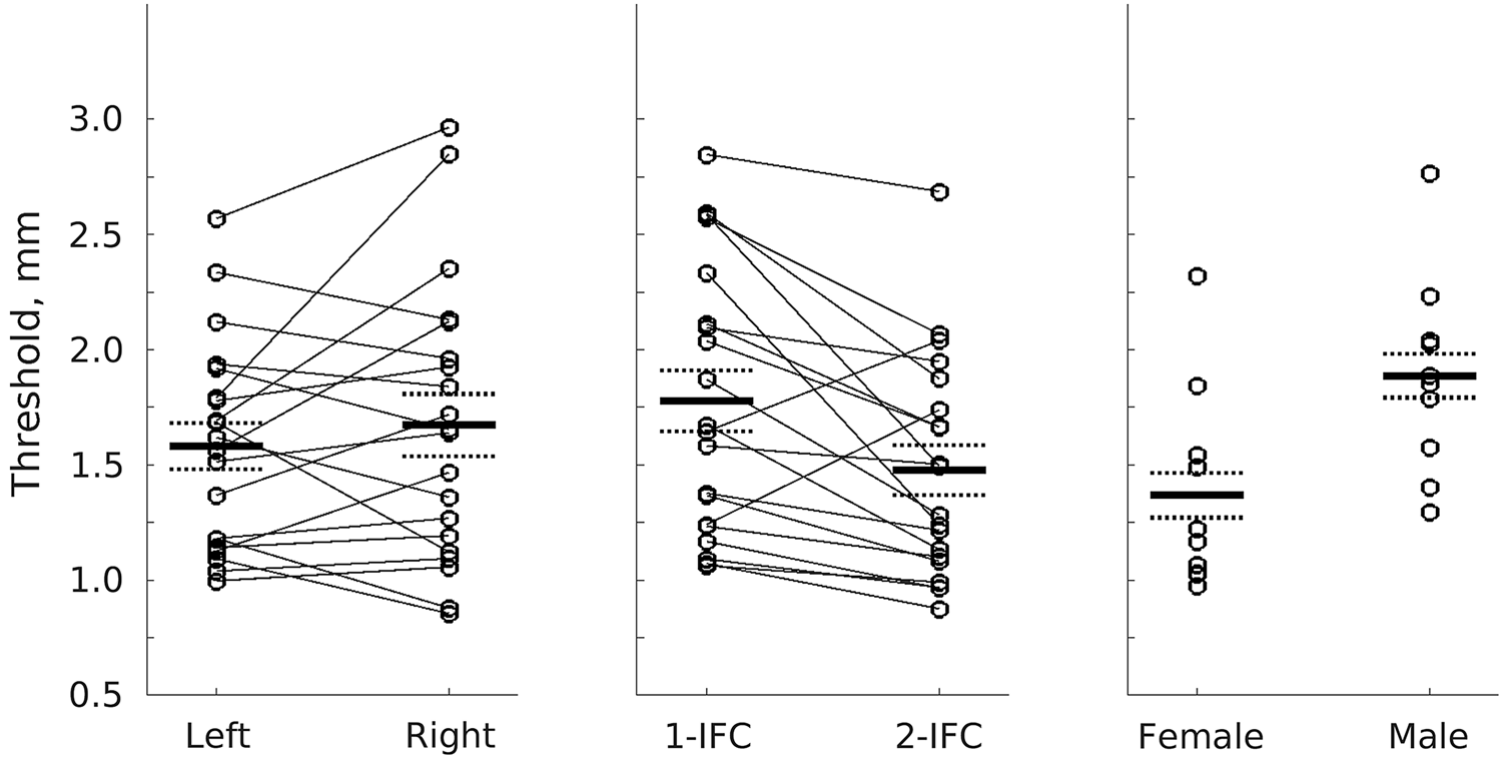
Grating orientation discrimination threshold depends on experimental design (1-IFC vs. 2-IFC) and participant sex (male vs. female), but not hand (left vs. right). Circles show individual data points; thin lines connect individual participants; thick black lines show the mean, broken lines show the mean ± the standard error of the mean

A significant effect of experimental design demonstrated that participants discriminated better between gratings in the two-interval design (M = 1.48, SD = 0.479, SE = 0.107 mm) than the one-interval design (M = 1.78, SD = 0.585, SE = 0.131 mm, M difference = 0.301, SD = 0.398, SE = 0.089 mm, *t*(19) = 3.39, *p* = 0.003, d = 0.758). Sex also influenced GOT performance with females (M = 1.37, SD = 0.436, SE = 0.138 mm) performing significantly better than males (M = 1.89, SD = 0.424, SE = 0.134 mm, M difference = -0.518, SD = 0.431, SE = mm, *t*(19) =   – 2.69, *p* = 0.014, *d* =  – 1.20). No differences were observed between left (M = 1.58, SD = 0.447, SE = 0.1 mm) and right hands (M = 1.67, SD = 0.604, SE = 0.135 mm, M difference =  – 0.0901, SD = 0.386, SE = 0.0863 mm, *t*(19) =  – 1.04, *p* = 0.309, *d* =  – 0.233). Figure 2 shows these results.

### Experiment 2: no effect of hand visibility or practice, but a significant effect of hand posture

**Fig. 3:**
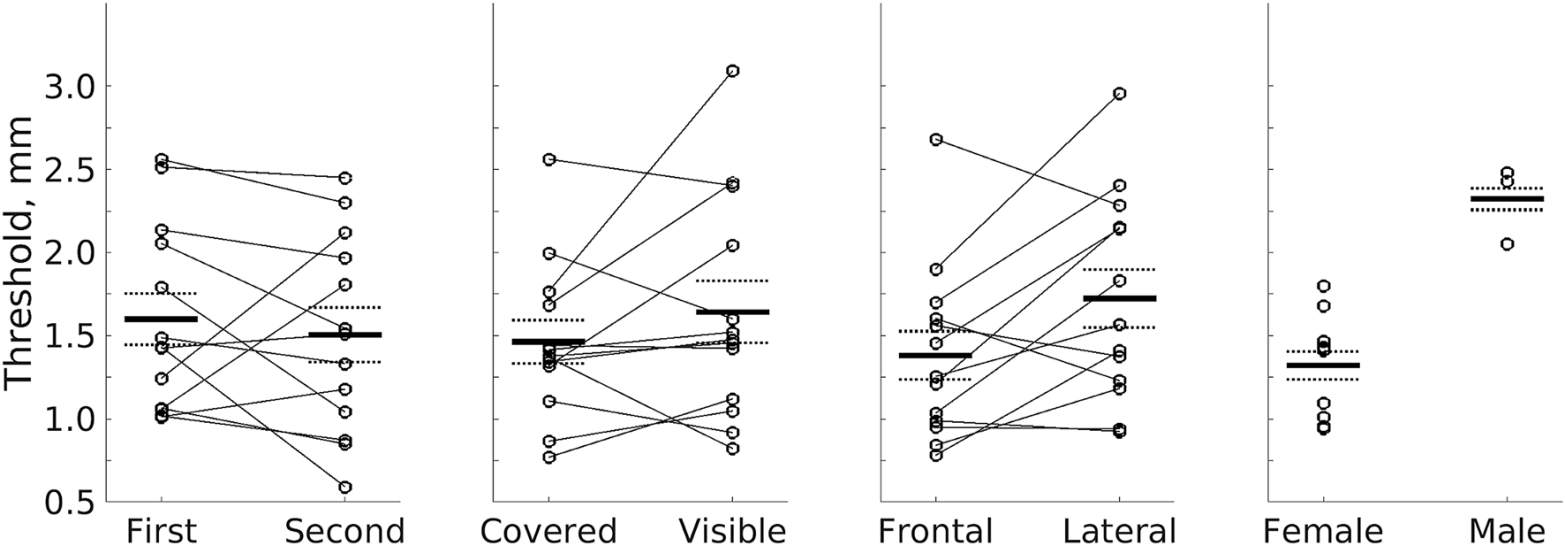
Grating orientation discrimination is affected by hand posture but not by hand visibility or block order. Mean thresholds are shown for blocks (first vs. second), visibility (covered vs. visible), hand position (frontal vs. lateral) and sex (male vs. female). Circles show individual data points; thin lines connect individual participants; thick black lines show the mean, broken lines show the mean ± the standard error of the mean

Contrary to previous reports of the VET, there was no effect of hand visibility, with similar performance when the hand was covered (M = 1.46, SD = 0.469, SE = 0.13 mm) and visible (M = 1.64, SD = 0.671, SE = 0.186 mm, M difference =  – 0.179, SD = 0.508, SE = 0.141 mm, *t*(12) =  – 1.27, *p* = 0.229, *d* =  – 0.352). However, a significant effect of hand position was observed—performance was better with the hand in front (M = 1.38, SD = 0.526, SE = 0.146 mm) compared to at the side (M = 1.72, SD = 0.627, SE = 0.174 mm, M difference =  – 0.341, SD = 0.505, SE = 0.14 mm, *t*(12) =  – 2.44, *p* = 0.031, *d* =  – 0.678). Since there were only three males in the sample of 13, we did not analyse the effect of sex, however females (M = 1.32, SD = 0.304, SE = 0.0961 mm) performed better than males (M = 2.32, SD = 0.234, SE = 0.135 mm). Since the participants performed each condition twice, we tested for practice effects. Averaged across all conditions, there was a small, non-significant improvement between the first (M = 1.60, SD = 0.559, SE = 0.155 mm) and second blocks (M = 1.50, SD = 0.595, SE = 0.165 mm, M difference = 0.095, SD = 0.498, SE = 0.138 mm, *t*(12) = 0.688, *p* = 0.505, *d* = 0.191). Figure 3 summarises the findings for these four factors.

### Experiment 3: effect of hand posture replicates

**Fig. 4:**
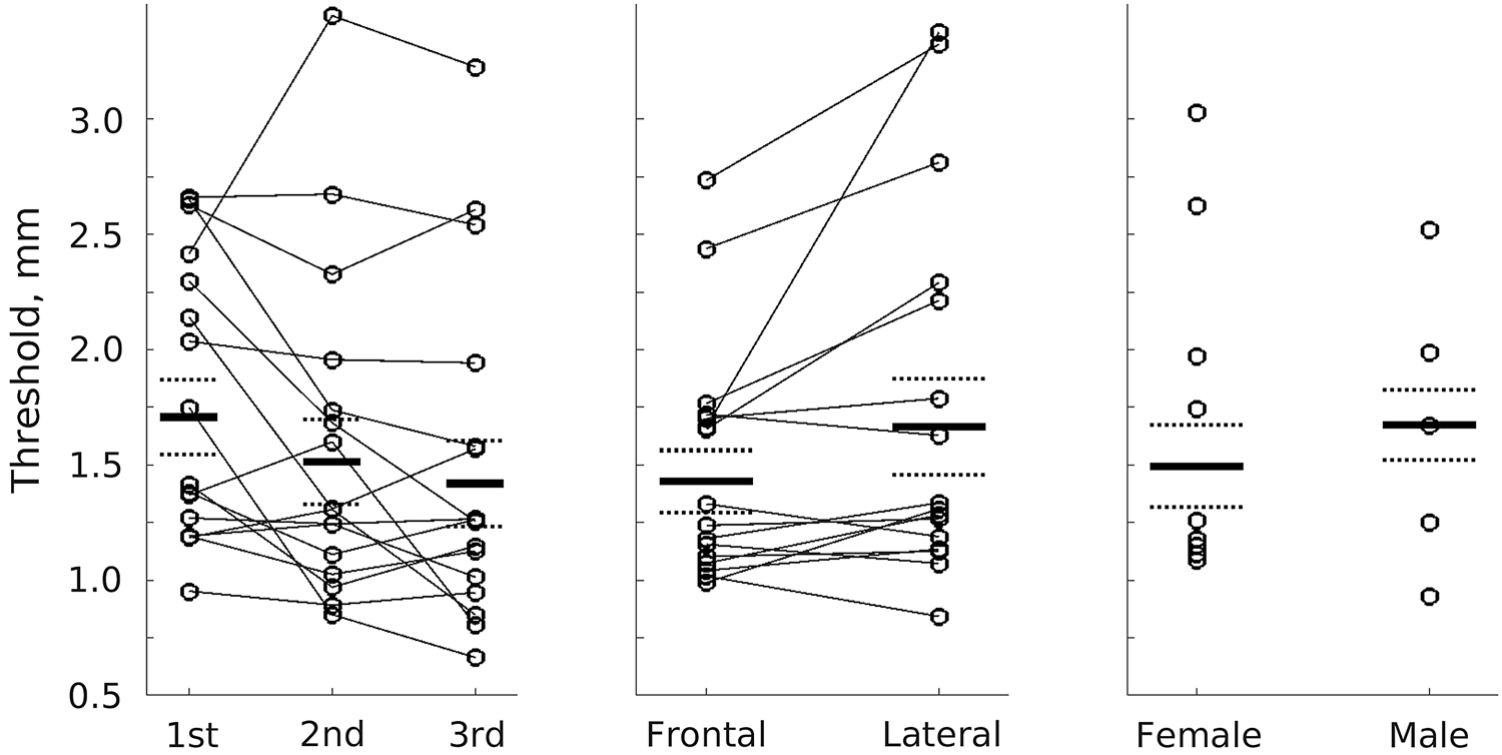
Grating orientation discrimination threshold is better when the hand is in a frontal rather than lateral posture, replicating the same effect in Experiment 2. Mean thresholds for blocks (first, second and third), hand position (frontal vs. lateral) and sex (male vs. female) are shown. Circles show individual data points; thin lines connect individual participants; thick black lines show the mean, broken lines show the mean ± the standard error of the mean

As in Experiment 2, there was a significant effect of hand position, with better performance for frontal (M = 1.43, SD = 0.557, SE = 0.135 mm) than for lateral postures (M = 1.66, SD = 0.862, SE = 0.209 mm, M difference =  – 0.237, SD = 0.458, SE = 0.111 mm, *t*(16) =  – 2.13, *p* = 0.049, *d* =  – 0.517). The effect of participant sex followed a similar direction to that in Experiments 1 and 2, with slightly better thresholds in the 12 females (M = 1.49, SD = 0.734, SE = 0.212 mm) than the 5 males (M = 1.67, SD = 0.622, SE = 0.278 mm). There was a significant practice effect, with performance improving from the first (M = 1.71, SD = 0.668, SE = 0.162 mm), to the second (M = 1.51, SD = 0.755, SE = 0.183 mm) and third blocks (M = 1.42, SD = 0.763, SE = 0.185 mm, first vs. third block, M difference = 0.288, SD = 0.474, SE = 0.115 mm; one-way ANOVA across three blocks, F(2,32) = 4.15, *p* = 0.025). Figure 4 shows these results.

Across-experiment analyses: There were no significant differences between overall performance in Experiments 1 (M = 1.63, SD = 0.496, SE = 0.111 mm) and 2 (M = 1.55, SD = 0.520, SE = 0.144 mm, *t*(31) = 0.417, *p* = 0.680), 1 and 3 (M = 1.55, SD = 0.689, SE = 0.167 mm, *t*(35) = 0.414, *p* = 0.681), or 2 and 3, *t*(28) = 0.025, *p* = 0.980. A random-effects meta-analysis in JASP 0.9.2 of the effect of hand posture across Experiments 2 and 3 gave a combined effect size of M = 0.277, SE = 0.087 mm (Z = 3.19, *p* = 0.001), with better performance with the hand in front than at the side. A similar analysis of the effect of sex across all three experiments found that females were better than males by M = 0.631, SE = 0.229 mm (Z = 2.75, *p* = 0.006).

Meta-analysis: Across 27 relevant studies, we found 50 relevant independent effect sizes. 47 contained sufficient information to compute or estimate Cohen’s d. An overall meta-analysis revealed a significant benefit of non-informative visual input over viewing a neutral object, covered hand, or when blindfolded (Cohen’s *d* = 0.538, SE = 0.747, Z = 7.20, *p* < 0.001). There was significant heterogeneity, and only weak evidence for a publication bias, in which some negative VET effects seemed to be ‘missing’ from the literature (Kendall’s tau = 0.197, *p* = 0.054; Egger’s sei = 1.17, *p* = 0.244). A trim-and-fill analysis identified seven ‘missing’ negative effect sizes, reducing the overall effect size (*d* = 0.440, SE = 0.77, Z = 5.75, *p* < 0.001). Including our new data from Experiment 2 reduced the overall effect size to 0.519 (SE = 0.076, Z = 6.85, *p* < 0.001) and after trim-and-fill to 0.430 (SE = 0.079, Z = 5.44, *p* < 0.001). Some of the between-study heterogeneity may arise from the type of task performed: while tasks based on ‘spatial’ (*d* = 0.587, SE = 0.086, Z = 6.86, *p* < 0.001) and ‘intensity’ (*d* = 0.524, SE = 0.155, Z = 3.38, *p* < 0.001) judgements produced significant VET effects, those based on ‘temporal’ judgements did not (*d* = 0.277, SE = 0.254, Z = 1.09, *p* = 0.277). Including only the grating orientation tasks, as used in our own work, resulted in a significant VET (*d* = 0.570, SE = 0.179, Z = 3.17, *p* = 0.002). Overall, the meta-analysis revealed a VET effect size, Cohen’s d, of between 0.43 and 0.54. Several limitations of this and the previous meta-analysis (Eads et al. 2015) are discussed in Supplementary Materials.

## Discussion

Three experiments aimed first to validate our experimental apparatus and second to investigate visual and postural factors affecting tactile perception in the GOT. Across all three experiments, thresholds ranged from around 0.75 to 3 mm, with mean thresholds around 1.5 mm. This is comparable to thresholds found in previous studies (Johnson and Phillips 1981; Peters et al. 2009). Most of the participants had not done the GOT before, and received only one or two short practice blocks. No doubt with further training and practice (Johnson and Phillips 1981), the participants would improve, as suggested by the significant effect of block number in Experiment 3.

The effect of experimental design—one versus two intervals—was expected. Discrimination accuracy improves when two opportunities are given to perceive a stimulus—making a relative rather than an absolute judgement. Since we used the same 76% correct criterion for both, it is unsurprising that thresholds were higher in the one interval case. This manipulation was done partly as a ‘sanity check’, and given that mean thresholds in the one interval task were around 1.75 mm, it allowed us to use the (faster) one-interval design for Experiments 2 and 3.

The effect of sex was also expected. Previous reports found that females tend to have lower thresholds than males, and that this is explained by finger size alone (Peters et al. 2009). Experiment 1 was designed to test for this effect, with ten males and ten females recruited. Experiments 2 and 3 did not try to recruit equal numbers of males and females, but the effect of sex remained in the same direction, and a meta-analysis across all our data confirmed this effect of sex. In hindsight, we should have measured participants’ hand and finger sizes to better account for this effect. People with smaller fingers typically have a higher density of Merkel cells in the fingertip ridges (Dillon et al. 2001), enabling them to identify gratings of a smaller width compared to those with larger fingers.

Having validated our experimental apparatus, we then investigated the effects of hand visibility and hand posture on GOT thresholds. Interestingly, no effect of visibility was found, which failed to replicate the VET observed in previous studies (Taylor-Clarke et al. 2004; Eads et al. 2015). The proposed VET effect suggests that non-tactile sources of information such as viewing the hand affect tactile spatial acuity. The VET has been reported using a number of experimental tasks, including the GOT, but also the two-point discrimination task. While the GOT provides a reliable measure of tactile spatial acuity, the two-point discrimination task does not. For example, Johnson and Phillips (1981) trained participants on four spatial acuity tasks. On the two-point discrimination task, they found that well-trained participants could distinguish two points when they were 0 mm apart, corresponding to impossibly high (i.e. infinite) spatial acuity. Johnson and Phillips (1981) concluded that the two-point task contains non-spatial confounds such as intensity, overall stimulus magnitude, and the number of edges in the stimulus. By contrast, highly trained participants achieved GOT thresholds at the fingertip of around 1 mm.

One explanation of our failure to replicate the VET is that the GOT may be less susceptible to visual or higher-level cognitive influences than other tactile tasks, such as the two point discrimination task. Perhaps the VET effect does not specifically improve tactile spatial resolution, but rather acts on a different aspect of touch? Another explanation is that we were simply unlucky—we only tried to replicate the VET effect in one experiment, and our post hoc meta-analysis shows that our experiment, like many others, only had 44% power to detect the likely VET effect size. Our meta-analysis suggests that the second explanation is likely—of 11 reviewed experiments using the GOT, 3 found negative VET effects and 8 positive, with a significant overall meta-analytic effect size, and no evidence of publication bias. Both our sample size (14) and numbers of trials per condition (45–135) are comparable to those used in the remaining literature (medians = 12 and 40, respectively). One further possibility is that we did not find a VET effect because participants in our study were not looking specifically at the skin surface stimulated (i.e. the fleshy side of the index finger), but rather at the back of the hand. While this remains a possibility, we note that, of the 50 effect sizes included in our meta-analysis, 24 did not find a significant VET effect, including 2 where the stimulated skin surface was viewed (Fiorio and Haggard 2005; Longo et al. 2011). Further, several studies removed visual feedback by turning off the lights when the tactile stimuli were delivered (e.g. Fiorio and Haggard 2005; Cardini et al. 2012; Konen and Haggard 2014), and others who reported significant VET effects also did not require participants to view the stimulated skin surface directly (e.g. Forster and Eimer 2005; Sambo et al. 2009). Further work may be needed to distinguish why some studies found the VET effect and others did not.

The effect of hand position on tactile spatial acuity was more successful, replicated in Experiment 3, and survived an across-experiment meta-analysis. The improved performance when the hand was positioned in alignment with the direction of the eyes, head, and body, and with the direction of the stimulus diagrams shown to the participant (i.e. a posturally congruent condition; Fig. 1D) suggests that tactile spatial perception might use an ‘eye-centred’ (or head-, body-, or world-centred) rather than a ‘hand-centred’ reference frame. This result may reflect the importance of proprioception acting as a mediator for tactile performance. The effect may also reflect the comfort of or participants’ experience with the two hand postures.

There are a few limitations to our work. First, the method of tactile application was different in Experiment 1 (manual) from Experiments 2 and 3 (automatic), limiting the generalisation of findings across our studies. While consistency across studies was sought and the experimenters applied the stimuli as similarly as possible, differences in application could have affected the timing, pressure and orientation of the gratings, which may have impacted performance. Against this possibility, mean thresholds were not significantly different between the three experiments. Second, we did not measure the force with which the gratings pressed into the participants’ finger pads, partly due to lack of hardware or expertise. It is possible that different hand postures could have resulted in different forces applied to the finger tips, for example if the fingertip in one posture pushed further into the aperture than another. We do not have any data with which to assess this possibility. Third, the participants’ hand and finger sizes were not measured. We assume that the sex differences observed in this study were due to differences in hand and/or finger size, but this was not explicitly measured. Fourth, we did not conduct a priori power analyses to determine the sample sizes for our experiments. Rather, the number of participants was determined by convenience (10 males, 10 females in E1; 14 and 18 participants in E2 and E3, respectively). Future studies should use the meta-analytic estimates of effect size that we have provided, and run experiments to achieve 80% statistical power. Across all the evidence that we have assessed, we estimate that the VET has a Cohen’s d of about 0.5. Future studies should therefore test at least 32 participants to achieve 80% power to detect a two-sided effect of the visual enhancement of touch.

## Supplementary Information

Below is the link to the electronic supplementary material.

Supplementary file1 (DOC 47 KB)

Supplementary file2 (XLSX 90 KB)

## Data Availability

All raw data, analytic code, 3D-printing files for the grating stimuli, and a detailed meta-analysis of 27 previous studies are freely available for re-use at our OSF project page: https://osf.io/da893/.
