# Supplementary material for: Hand posture, but not vision of the hand, affects tactile spatial resolution in the grating orientation discrimination task
Source: Exp Brain Res. 2022 Sep 8;240(10):2715–23. doi: 10.1007/s00221-022-06450-3 (PMC9510114; doi:10.1007/s00221-022-06450-3)
Supplement: Supplementary file 1 — Supplementary file1 (DOC 47 KB) [file 221_2022_6450_MOESM1_ESM.doc]

**French, Di Chiaro, & Holmes**

**Supplementary Materials: Non-systematic review and meta-analysis of relevant studies and their VET effect sizes**

A full list of studies and effects assessed is provided in the Supplementary spreadsheet and at our OSF page: <https://osf.io/da893/>. The following provides a discussion of several papers and effect-sizes that were difficult to assess, or contained statistical problems that required re-calculation of effect-sizes.

**Previous meta-analysis**

A meta-analysis of the effect of vision on tactile acuity was reported by Eads and colleagues (2015). We have re-done this analysis for several reasons, explained here.

*1. Important studies and experiments were missed*

Eads and colleagues assessed 432 articles and searched for others via the references to result in 10 studies. They focussed on the healthy adult population with a visual manipulation (view body, view control condition) and a measure of tactile acuity. They excluded non-English studies, as well as those in which a virtual or mirror-image of the body was provided.

Despite these inclusion criteria, important data were missed. For example, Eads and colleagues included one experiment (E4) from Press et al. (2004) which found a significant VET effect in a spatial reaction time discrimination task. But they missed the almost-identical experiment 3, which found a significant negative VET effect in the RT data. No reason for including E4, but excluding E3 was given. Indeed, 6 of the 7 VET effect sizes reported by Press et al. (2004) were negative, but only the single positive VET effect was included in the meta-analysis.

*2. Tactile ‘acuity’ was loosely defined*

The selection of studies for the meta-analysis by Eads et al. (2015) was for the paper’s assessment of ‘tactile acuity’. This criterion included studies using the two-point discrimination (2-PD) task, the grating orientation discrimination (GOD) task, tactile spatial discrimination tasks (i.e., one of two potential tactors are stimulated and participants reported which location it came from), and one study used amplitude detection and discrimination tasks. The 2-PD task is not a measure of tactile spatial resolution or acuity (Craig & Johnson 2000); the GOD can provide a reliable estimate of tactile acuity (Johnson & Phillips 1981); it is arguable whether discriminating between two discrete vibration locations provides a measure of tactile acuity; detecting and discriminating vibrations at a single location on the skin can never provide a measure of tactile acuity. It seems that Eads et al.’s (2015) study this was not a systematic review of tactile ‘acuity’ at all, but was rather a broad but incomplete selection of studies of the VET.

*3. Effect-sizes were incorrectly calculated*

Eads and colleagues (2015) included in their Table 2 three studies selected as evidence for the VET effect on tactile acuity (Cardini et al. 2011, 2012, Taylor-Clarke et al. 2004). They reported the standardised mean differences (SMD) for these studies as 2.43, 1.48, and 7.24 respectively. It is not stated exactly what these SMD measures are (e.g., Cohen’s d, Hegdes g, or Glass’s delta), but we assume that Cohen’s d was used. If so, Cohen’s d values between 1.48 and 7.24 are extremely large effect-sizes. Looking more closely at Eads and colleauges’ (2015) meta-data, the authors reported much smaller standard deviations in the three studies than expected. For example, Cardini et al.’s (2011) data are reported with the *View hand* data having a mean of 75% and a SD of 2%, and the *View object* as a mean of 70% and a SD of 2%. We believe that these reports of SD are actually estimates of SE. From Cardini et al. (2011):

*“Judgments of grating orientation were significantly above chance both when viewing the hand (75% correct; t(14) = 15.61, P <0.0001) and the object (70% correct; t(14) = 11.22, P < 0.0001).” p 2016*

We can calculate the SE by using the differences between the reported accuracies (75%, 70%) and chance (50%), and dividing by the t-score (which is measured in units of SE):

Hand: SE = (75-50)/15.61 = 1.602

Object: SE = (70-50)/11.22 = 1.783

Both of these values for SE seem to have been rounded up to 2 and reported as SD by Eads et al. (2015). Instead, SDs can be calculated by multiplying the SE by the square-root of N:

Hand: SD = 1.602*√(15) = 6.203

Object: SD = 1.783*√(15)=6.904

Likewise, we computed Cohen’s d for this study using the reported t-statistic for the mean difference between conditions, from Cardini et al. (2011):

*“the difference between these conditions was significant, with performance being better when viewing the hand than when viewing the object (t(14) = 3.91; P < 0.01, 2-tailed; Fig. 2).” p 2016*

A t-score of 3.91 means that there were 3.91 standard errors between the mean difference between the two conditions, and zero, so:

SE = (75-70)/3.91 = 1.279

SD = SE*√(N) = 4.953

We calculate Cohen’s d for this experiment in Cardini et al. (2011) as the difference between means (5%), divided by the within-participants standard deviation of that difference, 4.953%:

Cohen’s d = (75%-70%)/4.953% = 1.01

Eads and colleagues (2015) reported the standardized mean difference, which we assume was intended to be Cohen’s d, as 2.43.

In summary, the previous meta-analysis of Eads and colleagues (2015) did not include all relevant studies, used a loose and arbitrary definition of tactile acuity, and seems to have mistaken SE for SD when calculating effect-sizes. For these reasons, a non-systematic review and meta-analysis was undertaken of all the evidence on the VET that we could find (27 studies and 50 independent effect-sizes) by following references from and to Eads et al.’s (2015) study. Several comments and points of discussion are included below, to put some of the meta-analysis data into context.

**One-tailed or two-tailed?**

All p-values in the supplementary table are two-tailed. Several studies relied on one-tailed p-values explicitly in their manuscript (Kennett et al 2001; Haggard et al 2007), or seem to have done so, by inference from other reported statistics (Serino et al 2009).

**Post-hoc & non-parametric tests**

In several cases, authors reported the critical comparisons for the VET effects as ‘post-hoc’ or non-parametric tests. These often came with no statistical values other than a p-value. In each case, the p-value was used to calculate a t-statistic in order to include the effect in the meta-analysis.

**Rounding and typographical errors**

Wherever possible, the statistical values reported in the text were used in the meta-analysis. Often, however, the most-precise values were used preferentially. For example, if a t-statistic was reported as t(32)=2.36, p<.05, the p-value was recomputed from t as p=.0195. Conversely, if the statistic was given as t(19)=2.2, p=.044, the more precise p-value was used to improve the t-value estimate to t(19)=2.16. When authors used unconventional or incomplete statistical reporting methods, for example giving only ‘p<.02’, this was assumed to be p=.02, and the t-statistic computed from that.

In one case, the mismatch between the reported t- and p-statistics was so large that some stronger assumptions needed to be made. Serino et al. (2009, p. 607) reported one p-value as “p<.04”, but this did not match its reported t-value, “t(1,9)=5.98”. Assuming that this was a typographical error, several alternative values where checked. A t-value of 1.98 with 9 degrees of freedom has a two-tailed p-value of .079, which fits with the (presumably one-tailed) p-value reported of “<.04”. This interpretation of the data gives a standard error of the effect size (0.273) which is very close to that of the other experiments in that paper (E1=0.307, E2=0.294). Only t-values between 1.76 and 1.82 give an SE within the range of these first two experiments. The t(9)=1.98 value and two-tailed p-value of .079 was therefore used as the most likely explanation for this (mis)reported effect size. The authors of this study did not report using a one-tailed test for this statistic.

**Statistical double-dips – selecting data biased the remaining samples**

In the paper by Serino et al. (2007), effect sizes had to be estimated based on combining two groups that had been split post-hoc into ‘lower’ and ‘higher’ performing participants. This post-hoc data-split constitutes a statistical ‘double-dip’, because the data used to split participants were included in subsequent analyses (i.e., the interaction between group and condition, as reported on p. 1104). This technique creates spurious ‘significant’ differences between conditions much more frequently than the traditional 5% alpha criterion. See Holmes et al. 2007, 2009a, 2009b for commentary on closely-related and similarly-flawed analysis strategies. The biased data were included in the meta-analysis by Eads et al. (2015).

In the paper by Fiorio & Haggard (2005), participants who did not show an absolute VET effect in the baseline (no TMS) conditions were removed from the analysis: 3 of 11 participants in Experiment 1 and 9 of 19 participants in Experiment 2. The authors acknowledged that it was ”not surprising” (p. 775) to find a significant VET effect when comparing these two baseline conditions previously used to select participants (i.e., comparing *View Hand no TMS* with *View Object no TMS*). But they did not report that this data selection and removal also biased all comparisons involving either of the two individual baseline conditions – the *View Hand no TMS* data were biased towards being higher than the true population mean, and the *View Object no TMS* data were biased lower than the true mean (Holmes 2007, 2009a, 2009b). The results of this paper relied upon comparisons between the two *View hand* conditions: the pre-selected *no TMS* baseline and the same condition with TMS over S1 (Experiment 1, Figure 2a; Experiment 2, Figure 2b). These results are a statistical double-dip. The only valid comparisons in the paper are therefore between conditions with TMS: In experiment 1 there was no significant VET effect with TMS over S1 (Cohen’s d=-0.100), and in Experiment 2 there was no significant VET effect with TMS over S1 (d=-0.849), or S2 (d=0.334), or when these two effects were averaged for the meta-analysis (d=-0.257). While it is possible that TMS over S1 or S2 changed the underlying VET effect, there is no valid evidence of that in this paper. Rather than ignoring this report completely, the VET effects under TMS were retained.

**References & Meta-analysis bibliography**

Cardini F, Longo MR, Haggard P (2011) Vision of the body modulates somatosensory intracortical inhibition. Cereb Cortex 21(9):2014–2022 DOI: 10.1093/cercor/bhq267

Cardini F, Longo MR, Driver J (2012) Rapid enhancement of touch from non-informative vision of the hand. Neuropsychologia 50(8):1954–1960 DOI: 10.1016/j.neuropsychologia.2012.04.020

Catley MJ, Tabor A, Miegel RG, Wand BM, Spence C, Moseley GL (2014) Show me the skin! does seeing the back enhance tactile acuity at the back? Man Ther 19(5):461–466 DOI: 10.1016/j.math.2014.04.015

Craig JC, Johnson KO (2000) The two-point threshold: Not a measure of tactile spatial resolution. Current Directions in Psychological Science, 9(1):29-32 DOI: 10.1111/1467-8721.00054

Eads J, Moseley GL, Hillier SL (2015) Non-informative vision enhances tactile acuity: a systematic review and meta-analysis. Neuropsychologia 75:179–185 DOI: 10.1016/j.neuropsychologia.2015.06.006

Fiorio M, Haggard P (2005) Viewing the body prepares the brain for touch: Effects of TMS over somatosensory cortex. Eur J Neurosci 22(3):773–777 DOI: 10.1111/j.1460-9568.2005.04267.x

Forster B, Eimer M (2005b) Vision and gaze direction modulate tactile processing in somatosensory cortex: Evidence from event-related brain potentials. Exp Brain Res 165(1):8–18 DOI: 10.1007/s00221-005-2274-1

Haggard P (2006) Just seeing you makes me feel better: Interpersonal enhancement of touch. Soc Neurosci 1(2):104–110 DOI: 10.1080/17470910600976596

Haggard P, Christakou A, Serino A (2007) Viewing the body modulates tactile receptive fields. Exp Brain Res 180:187–193 DOI: 10.1007/s00221-007-0971-7

Halligan PW, Marshall JC, Hunt M, Wade DT (1997) Somatosensory assessment: Can seeing produce feeling? J Neurol 244:199–203 DOI: 10.1007/s004150050073

Harris JA, Arabzadeh E, Moore CA, Clifford CWG (2007) Noninformative vision causes adaptive changes in tactile sensitivity. J Neurosci 27(27):7136–7140 DOI: 10.1523/JNEUROSCI.2102-07.2007

Holmes NP (2007) The law of inverse effectiveness in neurons and behaviour: Multisensory integration versus normal variability. Neuropsychologia 45(14):3340–3345 DOI: 10.1016/j.neuropsychologia.2007.05.025

Holmes NP (2009a) Inverse effectiveness, multisensory integration, and the bodily self: Some statistical considerations. Conscious Cognit 18(3):762–765 DOI: 10.1016/j.concog.2009.04.009

Holmes NP (2009b) The principle of inverse effectiveness in multisensory integration: Some statistical considerations. Brain Topog 21(3-4):168–176 DOI: 10.1007/s10548-009-0097-2

Honoré J (1982) Posture oculaire et attention sélective à des stimuli cutanés. Neuropsychologia 20(6):727–730 DOI: 10.1016/0028-3932(82)90075-6

Honoré J, Bordeaud'hui M, Sparrow L (1989) Reduction of cutaneous reaction time by directing eyes towards the source of stimulation. Neuropsychologia 27(3):367–371 DOI: 10.1016/0028-3932(89)90025-0

Johnson KO, Phillips JR (1981) Tactile spatial resolution. I. Two-point discrimination, gap detection, grating recognition, and letter recognition. J Neurophysiol 46(6):1177–1191 DOI: 10.1152/jn.1981.46.6.1177

Kennett S, Taylor-Clarke M, Haggard P (2001a) Noninformative vision improves the spatial resolution of touch in humans. Curr Biol 11(15):1188–1191 DOI: 10.1016/S0960-9822(01)00327-X

Konen CS, Haggard P (2014) Multisensory parietal cortex contributes to visual enhancement of touch in humans: a single-pulse tms study. Cereb Cortex 24(2):501–507 DOI: 10.1093/cercor/bhs331

Larmande P, Cambier J (1981) L'Influence de l'etat d'activation hemispherique sur le phenomene d'extinction sensitive chez 10 patients atteints de lesions hemispheriques droites [Effect of the state of activation of the cerebral hemispheres on sensory extinction. A study on 10 patien. Revue Neurol 137:285–290

Leo F, Nataletti S, Brayda L (2020) Non-informative vision improves spatial tactile discrimination on the shoulder but does not influence detection sensitivity. Exp Brain Res 238(12):2865–2875 DOI: 10.1007/s00221-020-05944-2

Longo MR, Pernigo S, Haggard P (2011) Vision of the body modulates processing in primary somatosensory cortex. Neurosci Lett 489(3):159–163 DOI: 10.1016/j.neulet.2010.12.007

Newport RW, Rabb B, Jackson SR (2002) Noninformative vision improves haptic spatial perception. Curr Biol 12(19):1661–1664 DOI: 10.1016/S0960-9822(02)01178-8

Pierson JM, Bradshaw JL, Meyer TF, Howard MJ, Bradshaw JA (1991) Direction of gaze during vibrotactile choice reaction time tasks. Neuropsychologia 29(9):925–928 DOI: 10.1016/0028-3932(91)90056-E

Press CM, Taylor-Clarke M, Kennett S, Haggard P (2004) Visual enhancement of touch in spatial body representation. Exp Brain Res 154(2):238–245 DOI: 10.1007/s00221-003-1651-x

Sambo CF, Gillmeister H, Forster B (2009) Viewing the body modulates neural mechanisms underlying sustained spatial attention in touch. Eur J Neurosci 30(1):143–150 DOI: 10.1111/j.1460-9568.2009.06791.x

Serino A, Pizzoferrato F, Làdavas E (2008a) Viewing a face (especially one's own face) being touched enhances tactile perception on the face. Psychol Sci 19(5):434–438 DOI: 10.1111/j.1467-9280.2008.02105.x

Serino A, Padiglioni S, Haggard P, Làdavas E (2009) Seeing the hand boosts feeling on the cheek. Cortex 45(5):602–609 DOI: 10.1016/j.cortex.2008.03.008

Serino A, Farnè A, Rinaldesi ML, Haggard P, Làdavas E (2007) Can vision of the body ameliorate impaired somatosensory function? Neuropsychologia 45(5):1101–1107 DOI: 10.1016/j.neuropsychologia.2006.09.013

Taylor-Clarke M, Kennett S, Haggard P (2004) Persistence of visual-tactile enhancement in humans. Neurosci Lett 354(1):22–25 DOI: 10.1016/j.neulet.2003.09.068

Taylor-Clarke M, Kennett S, Haggard P (2002) Vision modulates somatosensory cortical processing. Curr Biol 12(3):233–236 DOI: 10.1016/S0960-9822(01)00681-9

Tipper SP, Lloyd DM, Shorland B, Dancer C, Howard LA, McGlone FP (1998) Vision influences tactile perception without proprioceptive orienting. NeuroReport 9(8):1741–1744 DOI: 10.1097/00001756-199806010-00013

Tipper SP, Phillips N, Dancer C, Lloyd DM, Howard LA, McGlone FP (2001) Vision influences tactile perception at body sites that cannot be viewed directly. Exp Brain Res 139(2):160–167 DOI: 10.1007/s002210100743

Whiteley L, Kennett S, Taylor-Clarke M, Haggard P (2004) Facilitated processing of visual stimuli associated with the body. Perception 33(3):307–314 DOI: 10.1068/p5053
